# Computational Insights Into Antimicrobial Peptide‐Enhanced Dental Resin Composites: Targeting *Porphyromonas gingivalis* Heme‐Binding Proteins and Biofilms

**DOI:** 10.1002/mbo3.70184

**Published:** 2025-12-01

**Authors:** Ravinder S. Saini, Doni Dermawan, Abdulkhaliq Ali F. Alshadidi, Rayan Ibrahim H. Binduhayyim, Rajesh Vyas, Fahad Hussain Alhamoudi, Sunil Kumar Vaddamanu, Mohamed Saheer Kuruniyan, Lujain Ibrahim N. Aldosari, Artak Heboyan

**Affiliations:** ^1^ Department of Allied Dental Health Sciences COAMS King Khalid University Abha Saudi Arabia; ^2^ Applied Biotechnology, Faculty of Chemistry Warsaw University of Technology Warsaw Poland; ^3^ Department of Prosthodontics, College of Dentistry King Khalid University Abha Saudi Arabia; ^4^ Department of Research Analytics, Saveetha Dental College and Hospitals, Saveetha Institute of Medical and Technical Sciences Saveetha University Chennai India; ^5^ Department of Prosthodontics, Faculty of Stomatology Yerevan State Medical University after Mkhitar Heratsi Yerevan 0025 Armenia; ^6^ Department of Prosthodontics, School of Dentistry Tehran University of Medical Sciences Tehran Iran

**Keywords:** antimicrobial peptides, dental resin composites, heme‐binding proteins, molecular docking, molecular dynamics, porphyromonas gingivalis

## Abstract

The research aimed at investigating the antibacterial potential of dental resin composites when combined with various antimicrobial peptides (AMPs) against *Porphyromonas gingivalis* heme‐binding proteins, which are associated with biofilm‐related infections in restorative dentistry. A multistage computational approach was implemented to assess the AMP interactions. Molecular docking analyses demonstrated the promising binding of resin constituents with AMPs, and Pardaxin exhibited the highest binding affinity, followed by Tachystatin and Thermolysin. The best performing AMPs were then docked with *P. gingivalis* heme‐binding proteins, and the complexes were subjected to 100 ns molecular dynamics simulations for stability assessment. The simulations confirmed stable interactions, while MM/PBSA binding energy calculations demonstrated significant binding strengths, particularly for Pardaxin (ΔG = −65.58 kcal/mol) and Tachystatin (ΔG = −48.71 kcal/mol), with Thermolysin also showing promising results (ΔG = −39.92 kcal/mol). The comprehensive analysis indicates the potential of incorporating Pardaxin, Tachystatin, and Thermolysin into dental resin composites to enhance their antibacterial activity against *P. gingivalis*. However, the study is limited to in silico assessments and relies on static representations of resin monomers that may not accurately represent the biological and clinical environment. Experimental validation through in vitro and in vivo studies, including cytocompatibility testing, peptide release behavior, and long‐term mechanical stability, is essential to establish their practical application in restorative dentistry.

## Graphical abstract

Computational analyses revealed that antimicrobial peptides Pardaxin, Tachystatin, and Thermolysin strongly bind to dental resin composites and Porphyromonas gingivalis heme‐binding proteins, enhancing antibacterial and anti‐biofilm potential. The study highlights AMP‐functionalized composites as promising next‐generation restorative materials with superior bioactive performance.

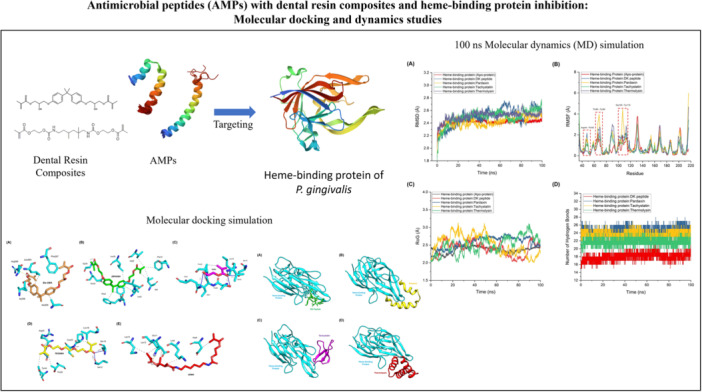

**Abbreviations**
Bis‐GMA: bisphenol A glycidyl methacrylate
EBPADMA: ethoxylated bisphenol A dimethacrylate
HADDOCK: high ambiguity‐driven protein‐protein docking
HEMA: 2‐Hydroxyethyl methacrylate
MD: molecular dynamics
MM/PBSA: molecular mechanics/poisson‐boltzmann surface area
NPT: number of particles, pressure, and temperature
NVT: number of particles, volume, and temperature
OPLS‐AA/L: optimized potentials for liquid simulations
PDB: protein data bank
PRODIGY: PROtein binDIng enerGY prediction
RMSD: root mean square deviations
RMSF: root mean square fluctuation
RoG: radius of gyration
SPCE: single point charge extended
TEGDMA: triethylene glycol dimethacrylate
UDMA: urethane dimethacrylate

## 1 Introduction

Restorative dentistry relies on dental resin composites because they combine esthetic appeal with easy application and versatility. Dental materials serve mainly as cavity fillings while also repairing chipped teeth and acting as bonding agents in dental treatments (Cho et al. 2021; Mulla et al. 2023). The synthetic resin matrix of dental resin composites made from Bis‐GMA or UDMA contains inorganic filler particles such as silica or glass to enhance their mechanical strength (Bhattacharya and Seong 2013; Pratap 2019a). The widespread application of these composites faces challenges from bacterial colonization and biofilm growth, which can lead to secondary caries and other oral diseases (Nedeljkovic et al. 2017; Chenicheri et al. 2017). Bacteria form biofilms that attach to surfaces and are covered by their natural extracellular matrix (ECM). The oral cavity biofilms host various bacterial species, including *Porphyromonas gingivalis*, which is identified as a primary pathogen responsible for periodontal diseases (Radaic and Kapila 2021; Gerits et al. 2017). *Porphyromonas gingivalis* employs gingipains, hemagglutinins, and heme‐binding proteins along with additional virulence factors to attach to host tissues and invade them while avoiding immune detection and acquiring iron from heme (Lamont et al. 2018; Aleksijević et al. 2022).

Researchers have developed a strong interest in adding antimicrobial properties to dental resin composites to reduce biofilm development and prevent infections. Conventional methods rely on incorporating antibacterial substances such as chlorhexidine, silver nanoparticles, and quaternary ammonium compounds (Imazato 2003; Corrêa et al. 2015). Additives for dental resin composites face limitations, including potential cytotoxicity effects, changes in the mechanical characteristics of composites, and diminished antimicrobial effectiveness over time from active agent release and depletion (Kasraei et al. 2014). The antimicrobial properties of dental resin composites can be improved using antimicrobial peptides (AMPs), which have become promising candidates in this field. AMPs represent short AMPs found naturally or synthesized in laboratories, which show broad‐spectrum effectiveness against bacteria, fungi, and viruses (Chung et al. 2020; Tripathi et al. 2023). AMPs destroy microbes by breaking down cell membranes, causing cell lysis, followed by cell death. AMPs can control the host immune response and prevent biofilm development according to studies (Duarte‐Mata and Salinas‐Carmona 2023; Zhang et al. 2021). In this context, in silico computational design offers a powerful and cost‐effective approach to explore the molecular basis of AMP‐material and AMP‐pathogen interactions before experimental validation (Alotaiq and Dermawan 2025; Dermawan and Alotaiq 2025). By simulating molecular interactions through docking and molecular dynamics studies, researchers can predict the stability, binding affinity, and inhibitory potential of AMPs, thereby minimizing experimental trial‐and‐error and accelerating material optimization (Isa and Kappo 2025; Liu et al. 2025). This computational approach also helps identify promising AMP candidates and elucidate their mechanisms of interaction with both biomaterials and microbial proteins, which are difficult to observe experimentally. Therefore, the central research question of this study is: Can AMPs be effectively integrated into dental resin composites to enhance their antibacterial potential against *P. gingivalis* by targeting its heme‐binding proteins responsible for biofilm formation and virulence? To address this, we employed an in silico multistage strategy to evaluate AMP–resin composite binding interactions and AMP–protein inhibition potential.

The goal of this study was to computationally investigate the feasibility of integrating selected AMPs into dental resin composites and to evaluate their inhibitory potential against P. gingivalis heme‐binding proteins, key components responsible for bacterial virulence and biofilm formation. This study examined how selected AMPs interact with dental resin composites using molecular docking and dynamic simulations. This study focused on predicting how peptides attach to resin composites and their interaction patterns. Specifically, molecular docking was used to predict AMP affinity toward representative resin monomers (Bis‐GMA, UDMA, EBPADMA, and HEMA), while subsequent AMP–protein docking and MD simulations were performed to evaluate the molecular stability and binding free energy of AMP–heme‐binding protein complexes. The researchers measured the peptide binding efficiency inside the composite matrix and investigated the resulting changes in the material properties. The research team focused on identifying the mechanism by which these peptides obstruct heme‐binding proteins in *P. gingivalis*. The proteins maintain the bacterium's vital functions and pathogenic capabilities and represent optimal targets for antimicrobial therapies (Zhang et al. 2020; Kosno et al. 2022). Overall, this study provides a computational framework to guide the rational design of AMP‐functionalized dental composites with improved antibacterial and anti‐biofilm capabilities.

## 2 Methodology

### 2.1 Selection of Dental Resin Composites for Investigation

Dental resin composites were selected for a detailed study based on their common market formulations and properties. The selection process required an in‐depth assessment to confirm that the materials matched the study objectives well. The selected resin composites displayed specific properties that matched the study's focus on AMP interactions exactly. The research analyzed dental resin composites, which were detailed in Table 1.

**Table 1:** Chemical structure and density of selected dental resin composites.

Dental Resin Composite	Chemical Structure	Density (g/cm³)
Bisphenol A glycidyl methacrylate (Bis‐GMA)	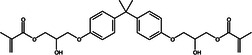	1.16
Ethoxylated bisphenol A dimethacrylate (EBPADMA)	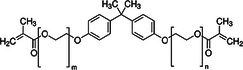	1.12
2‐Hydroxyethyl methacrylate (HEMA)	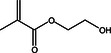	1.03
Triethylene glycol dimethacrylate (TEGDMA)	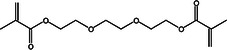	1.07
Urethane dimethacrylate (UDMA)	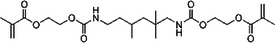	1.11

Dental resin composites were chosen for analysis due to their extensive application in restorative dental procedures and their specific chemical properties, which determine how they interact with AMPs. The dental material Bisphenol A Glycidyl Methacrylate (Bis‐GMA) possesses strong bonding properties and high viscosity, which provide essential durability (Pratap 2019b). Ethoxylated Bisphenol A Dimethacrylate (EBPADMA) was chosen because of its flexibility and decreased viscosity, which improves the workability of dental composites (Skrtic et al. 2006). 2‐Hydroxyethyl methacrylate (HEMA) was selected because of its hydrophilic properties, which enhance the adhesion and bonding strength (Van Landuyt et al. 2008). Triethyleneglycol Dimethacrylate (TEGDMA) was included for its function in cross‐linking, hence augmenting the mechanical strength of the composites (Comeau and Willett 2020). Urethane Dimethacrylate (UDMA) was chosen for its optimal mechanical quality and adaptability (Yang et al. 2024). The diverse properties of selected composites establish a comprehensive foundation for analyzing their behavior when combined with AMPs alongside their ability to enhance the antimicrobial properties of dental materials.

### 2.2 Selection of AMPs

The selection of AMPs followed exact criteria to validate the molecular docking and dynamics modeling processes. Only the PDB‐structured peptides were evaluated. Accurate structural data is essential for conducting effective docking research via modeling and simulation techniques. The SCOP database classified the chosen peptides as “peptides.” The peptides had a structural data resolution range between 0.5 and 2.5 Å. The active sites of the AMPs were examined using CASTp 3.0 (Tian et al. 2018). Supporting Data 1 contains the full data set, which includes the type of identification number from the Protein Data Bank (PBD ID), peptide sequence details, and information on the active residues of selected AMPs.

Based on these criteria, a set of 30 AMPs was chosen (Table 2), including Aurein, known for its broad‐spectrum antimicrobial activity and membrane‐disrupting ability (Liao et al. 2023); Cathelicidin, with potent antimicrobial properties and immune modulation (van Harten et al. 2018); Hepcidin, effective against various bacteria and involved in iron regulation (Nemeth and Ganz 2009); Pardaxin, recognized for its strong antimicrobial activity and membrane‐disrupting mechanisms (Bhunia 2010b); and Tachystatin, which has specific antimicrobial effects and interacts with bacterial cell walls (Fujitani 2002b). The selection of these peptides was based on their known antimicrobial qualities and existing high‐resolution structural data, which allow researchers to study how they interact with dental resin composites and evaluate their ability to prevent *P. gingivalis* heme‐binding proteins.

**Table 2:** The selected antimicrobial peptides (AMPs) based on the employed criterion.

Antimicrobial peptide	PDB ID	Active site (number of residues)	Ref
Aurein	1VM5	1, 2, 5	(Wang et al. (2005))
Beta‐defensin 2	1FD4	6, 7, 9, 10, 11, 12	(Hoover et al. (2000))
Bombinin	2AP7	1, 3, 4, 6, 7	(Zangger et al. (2008))
Cathelicidin	2K6O	1, 4, 5, 8	(Wang (2008))
Cecropin	1D9J	7, 9, 10, 11, 12	(Oh et al. (1999))
Chim2	8EB1	10, 11, 14, 15	(Viana de Freitas et al. (2023))
Dermcidin	2NDK	18, 19, 22, 25, 26, 29	(Nguyen et al. (2017))
Esculentin	5XDJ	2, 3, 6	(Loffredo et al. (2017))
Exendin‐4	3C59	26, 27, 28, 29, 32, 33	(Runge et al. (2008))
Hepcidin	3H0T	13, 14, 16, 18, 19, 20, 21, 22	(Jordan et al. (2009))
Hs05	6VLA	5, 8, 9	(Mariano et al. (2021))
Indolicidin	1HR1	9, 10, 11, 12, 13	(Friedrich et al. (2001))
Lactoferrin	1LFC	1, 23, 25	(Hwang et al. (1998))
Lavracin	2N8D	1, 3, 4, 6	(Pillong et al. (2017))
Magainin	2MAG	1, 2, 6, 9, 17, 21	(Gesell et al. (1997))
Melittin	2MLT	13, 16, 17, 20	(Terwilliger et al. (1982))
Microcin J25	4CU4	9, 10, 19, 20, 21	(Mathavan et al. (2014))
Nisin	1WCO	9, 12, 17, 19, 20, 21	(Hsu et al. (2004))
Pardaxin	2KNS	2, 3, 5, 6, 9, 15, 22, 23, 26, 27, 29, 30, 33	(Bhunia (2010a))
Piscidin	6PEZ	3, 4, 7	(Comert et al. (2019))
Pleurocidin	2LS9	10, 13, 14, 17, 20, 23, 24	(Amos et al. (2016))
Polyphemusin I	1RKK	7, 9, 12, 14	(Powers et al. (2004))
Protegrin‐1	1PG1	5, 6, 7, 14, 15, 16	(Fahrner et al. (1996))
PvHCt	2N1C	14, 15, 16, 17, 18, 19, 20, 22, 23	(Petit et al. (2016))
Subtilisin A	1PXQ	1, 4, 5, 7, 9, 10, 24, 25, 29, 30, 33	(Kawulka et al. (2004))
Tachyplesin‐1	2RTV	1, 2, 3, 16, 17	(Kushibiki et al. (2014))
Tachystatin	1CIX	5, 11, 12, 15, 18, 22, 23, 29	(Fujitani (2002a))
Temporin‐L	6GS5	4, 7	(Manzo et al. (2019))
Thanatin	8TFV	11, 13, 16, 17, 18	(Mandard et al. (1998))
Thermolysin	6FHP	258, 263, 267, 305, 306, 309, 310	(Fiebig et al. (2018))

### 2.3 Molecular Docking Simulations

This step included Molecular docking simulations, which were performed to assess the interactions between AMPs and dental resin composites, as well as their likelihood of binding to the heme‐binding protein of *P. gingivalis*. The simulations used the HADDOCK (High Ambiguity Driven protein‐protein DOCKing) stand‐alone version (Dominguez et al. 2003), recognized for its versatility and precision in investigating binding mechanisms and energetic interactions among biomolecules. Initially, AMPs were included in dental resin composites to identify those exhibiting the most advantageous binding relationships and performance. Subsequently, the top‐performing AMPs were docked to the heme‐binding protein of *P. gingivalis*. The X‐ray crystal structure of this target receptor, with a resolution of 1.80 Å, was retrieved from the Protein Data Bank (PDB ID: 3H8T (Wójtowicz et al. 2009)) with residue numbers 48, 78, 79, 80, 81, 122, 134, 148, 154, 164, and 173 as the active sites (determined using CASTp 3.0). The structure serves as a model for the heme‐binding protein's binding state while offering researchers a detailed guide to investigate potential inhibitory interactions. The DK peptide (sequence: DHYAVMISK) was used as a standard inhibitor. Researchers suggest this peptide inhibits heme‐binding proteins and holds potential as a treatment option for periodontal disease management (An et al. 2021; Yang et al. 2015). The primary objective of these simulations was to elucidate the binding modes of AMPs to heme‐binding proteins and assess their potential efficacy in disrupting protein function. PRODIGY (PROtein binDIng enerGY prediction was used to predict (Vangone and Bonvin 2015) the binding affinities of the AMP–resin and AMP–protein complexes. PRODIGY uses advanced computational techniques to estimate the binding affinity between protein‐ligand or protein‐protein interactions, which assists in selecting the most promising complexes for further experimental validation (Grassmann et al. 2024; Saini et al. 2025). All molecular docking simulations were executed on a high‐performance computing workstation with an Intel Core i7‐12650H processor, NVIDIA RTX 4060 graphics card with 8 GB of VRAM, and 16 GB of DDR5 RAM.

### 2.4 Molecular Dynamics (MD) Simulations

MD simulations helped study the stability and behavior of peptide‐protein complexes formed between AMPs and the heme protein of *P. gingivalis*. The study deployed GROMACS 2022.5 (Pronk et al. 2013) for MD simulations because of its acknowledged high performance and accuracy in biomolecular systems. All molecular simulations in this study were conducted using the Optimized Potentials for Liquid Simulations (OPLS‐AA/L) force field to model molecular interactions (Robertson et al. 2015). To properly accommodate the biomolecular complexes, the simulation box dimensions were established using the default cubic box settings (Alotaiq and Dermawan 2025). The simulation input files were prepared according to standard procedures, which included inserting counterions to preserve the system neutrality and water molecules using the Single Point Charge Extended (SPCE) model (Yuet and Blankschtein 2010). To remove steric differences and achieve system relaxation to a stable state, the steepest‐descent energy minimization approach was used. The system underwent a two‐phase equilibration. The phase 1 NVT ensemble equilibration enabled the system to stabilize conditions that balanced both the number of particles and volume and temperature. Phase 2 required system equilibration using the number of particles, pressure, and temperature (NPT) ensemble to sustain constant temperature and pressure levels. Following equilibration, scientists ran production MD simulations to observe protein‐protein complex dynamics for 100 ns. In this study, complex stability and conformational dynamics were evaluated by monitoring the RoG, root mean square deviation (RMSD), Root Mean Square Fluctuation (RMSF), potential energies, and hydrogen bonding interactions during simulations using molecular visualization tools, such as PyMOL (Schrödinger 2020) and UCSF Chimera (Pettersen et al. 2004). Key residues and intermolecular interactions within the anticipated protein‐protein complexes were inspected manually. These methods provide important insights into the processes governing the binding and stability of AMPs with heme‐binding proteins by enabling the analysis and interpretation of key structural features and interactions within simulated complexes.

### 2.5 Molecular Mechanics/Poisson–Boltzmann Surface Area (MM/PBSA) Calculations

The MM/PBSA method employs MD simulations to investigate peptide‐protein interactions involving AMPs with the heme‐binding protein of *P. gingivalis*. MD simulations produced a range of protein conformations, and representative snapshots were chosen for detailed analysis (Tian et al. 2014). Each snapshot was subjected to thorough energy computations encompassing gas‐phase energy calculations, solvation energy estimation using a continuum solvent model, and entropy calculations. These energy components were integrated to determine the binding free energy of the peptide‐protein complex (Yuan et al. 2024; Rifai et al. 2020). The gmx_MMPBSA module from the GROMACS simulation package allows us to perform these computations (Valdés‐Tresanco et al. 2021; Miller et al. 2012). This module enables users to compute the binding free energies of biomolecular complexes both precisely and efficiently. The MM/PBSA method is an effective tool for modeling the binding free energy of peptide‐protein interactions, which helps decode the energetic dynamics of biomolecular interactions (Panday and Alexov 2022). The following equation outlines the derivation process for the MM/PBSA binding free energy calculation.

ΔG_binding=ΔG_complex−ΔG_peptide−ΔG_protein,

where:

Δ*G*_binding: binding free energy associated with the formation of the protein‐protein complex.

Δ*G*_complex: free energy of the fully solvated protein‐protein complex.

Δ*G*_peptide: free energy of the peptide in its solvated state when unbound.

ΔG_protein: free energy of the protein in its solvated state when unbound.

The binding free energy was determined by calculating the difference between the free energy of the complex and the combined free energies of the unbound proteins. This calculation provided insights into the energetic alterations that occurred during the formation of the peptide‐protein complex, thereby elucidating the strength and stability of the interaction.

## 3 Results

### 3.1 Molecular Docking Simulations of AMPs and Dental Resin Composites

The research phase included molecular docking simulations to explore how AMPs could potentially interact with dental resin composites. The simulations evaluated several parameters, such as the HADDOCK score expressed in arbitrary units, together with binding energy, van der Waals energy, electrostatic energy, and desolvation energy across different component combinations. The research used computational simulations to evaluate both stability factors and binding affinities of AMP‐resin composite complexes to investigate their suitability as dental materials. Molecular docking simulations provided insights into the binding interactions and energetics of AMP‐resin composite complexes. Based on our research findings, we can identify the combinations that yield optimal interactions and show promise for incorporation into dental materials. According to the simulation results (AMPs), multiple molecular interactions form with resin composites. AMPs form molecular bonds with resin composites through both hydrophobic interactions and hydrogen bonds along with salt bridges. The combination of these molecular bridges resulted in enhanced stability and binding affinity for the complexes.

**Figure 1:**
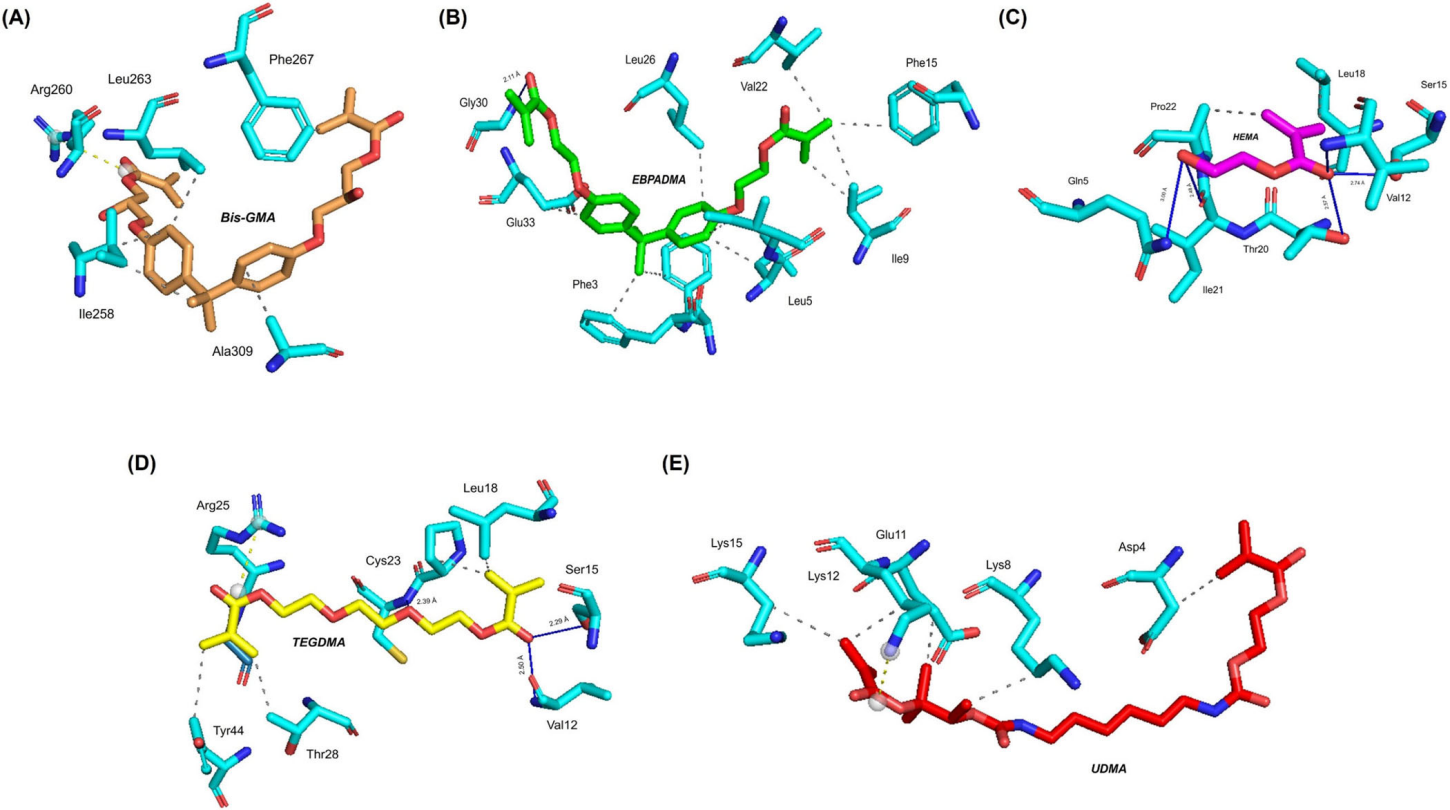
3D perspective of the most favorable complexes for each dental resin composite. (A) Bis‐GMA:Thermolysin complex. (B) EBPADMA:Pardaxin complex. (C) HEMA:Tachystatin complexes. (D) TEGDMA:Tachystatin complex. (E) UDMA:Cathelicidin complex.

The three‐dimensional perspective of the complexes that exhibited the greatest potential for each dental resin composite is shown in Figure 1. For example, the Bis‐GMA:Thermolysin complex exhibits five hydrophobic contacts and one salt bridge, which exemplifies a strong and consistent interaction between the constituents. A significant and specific binding affinity was observed in the EBPADMA:Pardaxin complex, as shown by the presence of 11 hydrophobic contacts and a hydrogen bond with Gly30 at a distance of 2.11. Similar to the previous example, the HEMA: Tachystatin complex formed five hydrogen bonds and two hydrophobic interactions with Gln5, Val12, Ser15, Thr20, and Ile21. The binding interface was stable and well defined because the bond lengths remained within the range of 1.99 to 3.00 Å. The TEGDMA:Tachystatin complex exhibited a total of four hydrophobic contacts, one salt bridge, and four hydrogen bonds with Val12, Ser15, Cys23, and Gly26. Four amino acids exhibited bond lengths between 2.29 and 2.77 Å. The material demonstrated a strong and uniform contact pattern that could lead to better performance. On the other hand, the UDMA:Cathelicin complex formed four hydrophobic interactions and one salt bridge, which demonstrated a consistent interaction that could improve the antibacterial function of the material. Table 3 presents the full results from the molecular docking simulations. The table displays binding energy values together with different energy components measured in kilocalories per mole (kcal/mol). The research findings enabled a quantitative evaluation of interactions between AMP‐resin composite complexes and supported comparative analysis of their stability and affinity. Supporting Data 1 contains the comprehensive findings of the molecular docking simulation.

**Table 3:** Molecular docking results of the top five dental resin composites:antimicrobial peptide complexes.

Resin composite:AMP complex	HADDOCK score (a.u.)	Binding energy (kcal/mol)	Van der Waals energy	Electrostatic energy	Desolvation energy	RMSD
**Bis‐GMA complexes**						
Bis‐GMA:Thermolysin	−34.1 + /− 1.5	−8.08	−21.0 + /− 1.2	−31.2 + /− 7.8	−10.4 + /− 0.4	0.3 + /− 0.0
Bis‐GMA:Tachystatin	−38.8 + /− 1.6	−7.98	−32.7 + /− 0.6	−17.0 + /− 4.0	−6.1 + /− 0.8	0.2 + /− 0.1
Bis‐GMA:Nisin	−35.6 + /− 0.7	−7.60	−25.5 + /− 1.2	−78.7 + /− 9.4	−2.3 + /− 0.5	1.6 + /− 0.1
Bis‐GMA:Pardaxin	−48.7 + /− 0.9	−7.59	−29.0 + /− 1.1	−1.9 + /− 2.1	−20.0 + /− 0.8	0.6 + /− 0.1
Bis‐GMA:Bombinin	−27.4 + /− 1.0	−7.57	−19.2 + /− 0.5	−67.5 + /− 4.1	−1.5 + /− 0.4	0.3 + /− 0.2
**EBPADMA complexes**						
EBPADMA:Pardaxin	−41.9 + /− 1.9	−8.14	−27.0 + /− 2.3	−30.1 + /− 10.6	−12.8 + /− 1.5	0.5 + /− 0.3
EBPADMA:Tachystatin	−35.8 + /− 1.0	−7.93	−27.6 + /− 1.2	−15.3 + /− 3.6	−7.1 + /− 1.0	0.3 + /− 0.2
EBPADMA:Thermolysin	−33.0 + /− 0.6	−7.87	−20.5 + /− 0.3	−20.9 + /− 6.0	−10.5 + /− 0.8	0.4 + /− 0.0
EBPADMA:Pleurocidin	−45.1 + /− 0.9	−7.64	−31.2 + /− 0.5	−30.1 + /− 11.3	−11.1 + /− 0.8	0.8 + /− 0.2
EBPADMA:Protegrin‐1	−28.4 + /− 0.7	−7.46	−23.2 + /− 0.9	−54.5 + /− 7.7	0.2 + /− 0.3	0.4 + /− 0.1
**HEMA complexes**						
HEMA:Tachystatin	−20.7 + /− 0.2	−6.24	−17.0 + /− 0.2	−27.1 + /− 0.8	−1.0 + /− 0.1	0.1 + /− 0.1
HEMA:Chim2	−19.2 + /− 0.4	−6.17	−13.2 + /− 0.7	−35.0 + /− 3.2	−2.6 + /− 0.5	0.3 + /− 0.2
HEMA:Cecropin	−18.6 + /− 1.0	−6.10	−13.8 + /− 0.7	−37.3 + /− 7.8	−1.0 + /− 0.4	0.4 + /− 0.2
HEMA:Thermolysin	−14.5 + /− 0.6	−6.03	−8.2 + /− 0.3	−1.5 + /− 0.6	−6.3 + /− 0.3	0.2 + /− 0.1
HEMA:Subtilisin A	−15.2 + /− 2.4	−5.98	−13.8 + /− 0.5	−26.1 + /− 4.6	−0.2 + /− 0.3	0.8 + /− 0.1
**TEGDMA complexes**						
TEGDMA:Tachystatin	−31.9 + /− 1.3	−6.90	−23.8 + /− 1.2	−46.4 + /− 7.1	−3.8 + /− 0.1	0.3 + /− 0.0
TEGDMA:Thermolysin	−25.9 + /− 1.1	−6.81	−15.9 + /− 0.5	−19.5 + /− 7.2	−8.2 + /− 0.2	0.2 + /− 0.1
TEGDMA:Beta‐defensin 2	−23.7 + /− 0.5	−6.77	−14.5 + /− 0.8	−68.0 + /− 3.4	−2.6 + /− 0.2	0.2 + /− 0.0
TEGDMA:Pardaxin	−30.4 + /− 1.1	−6.59	−17.0 + /− 0.9	−4.5 + /− 2.1	−13.6 + /− 1.1	0.4 + /− 0.2
TEGDMA:PvHCt	−25.5 + /− 0.8	−6.59	−15.7 + /− 0.9	−33.5 + /− 4.5	−8.7 + /− 0.6	0.7 + /− 0.1
**UDMA complexes**						
UDMA:Cathelicidin	−30.6 + /− 0.8	−7.42	−20.1 + /− 0.5	−96.0 + /− 8.6	−1.0 + /− 0.3	2.0 + /− 0.0
UDMA:Pardaxin	−38.6 + /− 0.6	−7.09	−22.0 + /− 1.2	−5.9 + /− 5.2	−16.5 + /− 1.7	0.5 + /− 0.1
UDMA:Pleurocidin	−34.4 + /− 1.3	−6.99	−21.2 + /− 0.6	−31.9 + /− 12.2	−10.8 + /− 1.1	1.4 + /− 0.1
UDMA:Chim2	−31.3 + /− 2.7	−6.86	−16.4 + /− 1.8	−26.9 + /− 4.0	−12.4 + /− 0.6	1.7 + /− 0.1
UDMA:Subtilisin A	−28.0 + /− 0.9	−6.85	−22.0 + /− 0.6	−47.3 + /− 5.2	−1.8 + /− 0.3	1.3 + /− 0.0

Molecular docking simulations were performed for multiple AMPs to evaluate their interaction capabilities with different dental resin composites. Pardaxin, Tachystatin, and Thermolysin ranked as the leading AMPs based on their docking scores and binding affinities, along with other interaction parameters. Pardaxin forms significant binding connections with various resin composite materials. Pardaxin showed a HADDOCK score of −48.7 ± 0.9 for Bis‐GMA resin composite, which included a binding affinity (Δ*G*) of −7.59 kcal/mol and a cluster size of 19 with an RMSD of 0.6 ± 0.1. The negative van der Waals energy contribution of −29.0 ± 1.1, combined with the electrostatic energy of −1.9 ± 2.1, demonstrates strong and stable binding interactions. Pardaxin's interaction with EBPADMA resulted in a HADDOCK score of −41.9 ± 1.9, and a binding affinity of −8.14 kcal/mol while forming a cluster size of 24 with an RMSD measurement of 0.5 ± 0.3. The buried surface area reached a notably high value of 854.2 ± 23.1, which demonstrates extensive contact with the resin surface. UDMA showed strong Pardaxin performance with a HADDOCK score of −38.6 ± 0.6 while having a binding affinity of −7.09 kcal/mol and an RMSD value of 0.5 ± 0.1 alongside a buried surface area of 794.5 ± 16.3.

Tachystatin also exhibited strong binding capabilities. Tachystatin bound to Bis‐GMA achieved a HADDOCK score of −38.8 ± 1.6 along with a binding affinity of −7.98 kcal/mol, and the cluster size was 11 with an RMSD of 0.2 ± 0.1. The van der Waals energy measurement of −32.7 ± 0.6 demonstrates strong hydrophobic interactions. EBPADMA demonstrated a HADDOCK score of −35.8 ± 1.0, a binding affinity of −7.93 kcal/mol, while presenting a cluster size of 15, and an RMSD of 0.3 ± 0.2. The buried surface area registered at 695.2 ± 18.3, which demonstrates effective contact with the resin composite. Tachystatin exhibited a HADDOCK score of −20.7 ± 0.2 and a binding affinity of −6.24 kcal/mol when paired with HEMA because of its extensive cluster size of 95 and RMSD of 0.1 ± 0.1. These data imply that tachystatin engages in stable and wide‐ranging interactions with HEMA.

Thermolysin demonstrated exceptional performance owing to its binding capabilities with bis‐GMA and various resin composites. Thermolysin achieved a HADDOCK score of −34.1 ± 1.5 when binding to Bis‐GMA and showed a binding affinity of −8.08 kcal/mol with a cluster size of 8 and an RMSD of 0.3 ± 0.0. Strong molecular binding interactions were revealed by the measured van der Waals energy of −21.0 ± 1.2, and an electrostatic energy measurement of −31.2 ± 7.8. Thermolysin produced a HADDOCK score of −33.0 ± 0.6, which showed a binding affinity of −7.87 kcal/mol for EBPADMA with a cluster size of 4 and an RMSD value of 0.4 ± 0.0. The buried surface area reached 621.8 ± 17.2, indicating a substantial interaction with the resin surface. Thermolysin showed a HADDOCK score of −25.9 ± 1.1 with TEGDMA and exhibited binding affinity of −6.81 kcal/mol while forming a cluster size of 6, which had an RMSD of 0.2 ± 0.1, thus demonstrating its versatile binding capacity. Detailed docking results are shown in Supporting Data 2.

**Figure 2:**
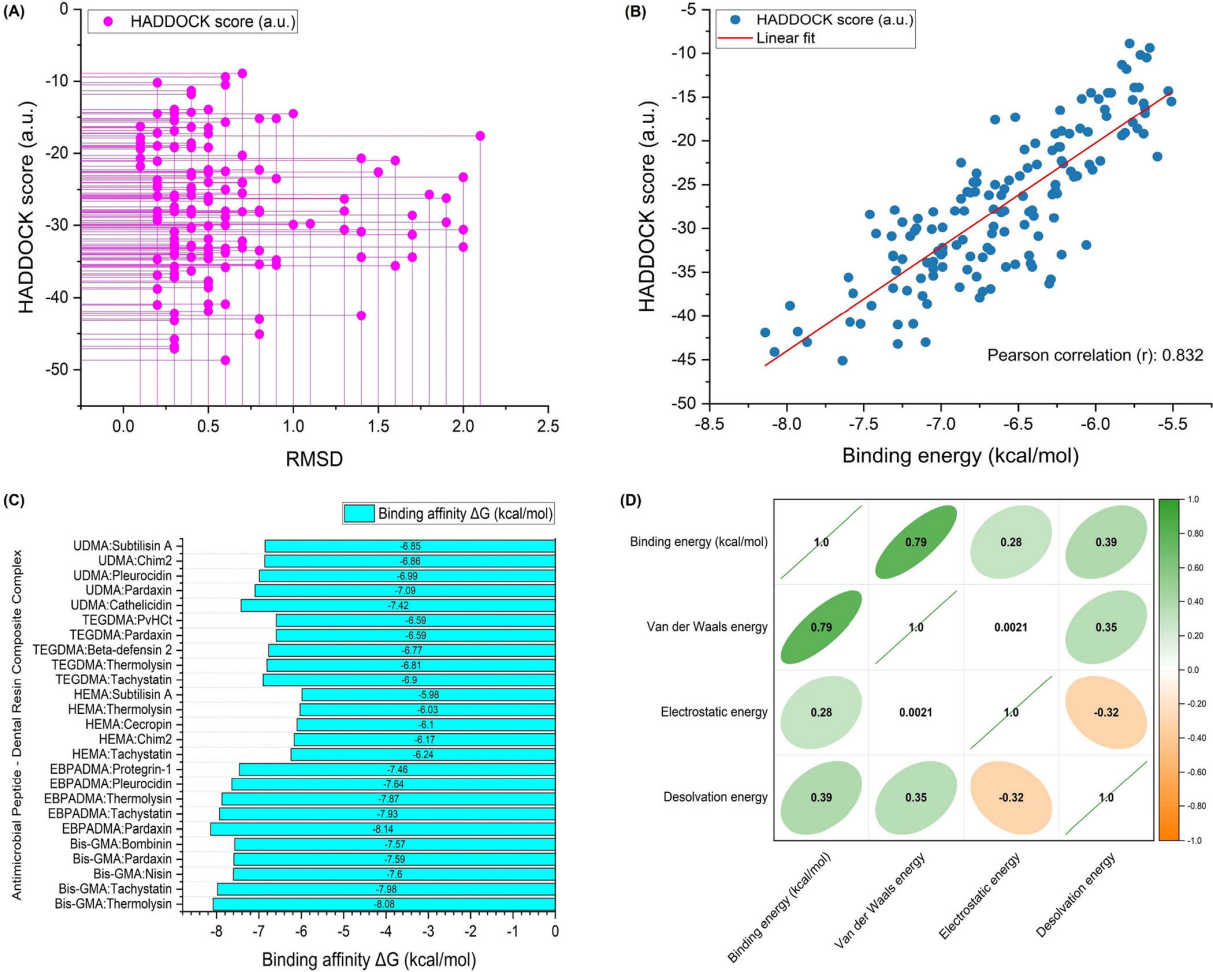
Molecular docking results overview. (A) Relationship between HADDOCK score and Root Mean Square Deviation (RMSD). (B) The correlation between HADDOCK score and binding affinity showed a strong Pearson correlation coefficient (r = 0.832). (C) Optimal resin composite:AMP complexes for each resin composite type, highlighting the lowest binding energy values indicative of a superior affinity. (D) Correlation matrix depicting the relationship between binding energy (kcal/mol) and individual energy components.

Molecular docking simulations produced highly favorable AMP‐resin pairings, which consistently showed RMSD values of less than 2.0 Å. A high degree of structural similarity between the predicted and optimal conformations was indicated by the fact that almost 86.67% of the combinations showed RMSD values below 1.0 Å. Figure 2A shows how the HADDOCK score corresponded to RMSD values. The RMSD value provides a measure of the structural differences between the predicted and optimal AMP‐resin complexes in this study. The plot illustrates that modifications in the HADDOCK score as a measure of binding affinity result in matching alterations in structural congruence between the predicted and optimal conformations according to RMSD measurements. A smaller RMSD value indicates that the predicted conformation matches the optimal conformation more closely, which means the binding interaction predictions are more accurate (Dermawan et al. 2019; Kufareva and Abagyan 2012). The observed correlation demonstrates how dependable docking simulations are used to forecast the beneficial interactions between AMP and resin molecules. Figure 2B shows a strong correlation between the HADDOCK score and binding affinity, based on a Pearson correlation coefficient of 0.832. The data coefficients confirm that these parameters exhibit a strong positive correlation. The near‐one value of the correlation coefficient confirmed a strong linear relationship between the HADDOCK score and the AMP‐resin binding affinity. Increased binding affinity manifested as higher HADDOCK score values according to the scoring system. Reduced HADDOCK scores indicated a weaker binding potential, while simultaneously showing decreased binding affinity. The strong correlation confirmed that the HADDOCK scoring system reliably and precisely predicted AMP‐resin complex binding affinities, demonstrating a strong alignment between the computational predictions and experimental data and validating the molecular docking methodology used in this study.

This study demonstrated significant differences in the binding affinities of various AMP‐resin complexes. The measurement of binding energy allows for a better understanding of the thermodynamic stability of the interactions between AMP and resin. Figure 2C shows the best AMP‐resin pairings for each dental resin type by highlighting the lowest binding energy values, which demonstrate strong binding interactions. The correlation matrix in Figure 2D demonstrates how the binding energy connects to separate energy elements, including the van der Waals energy, electrostatic energy, and desolvation energy, through graphical visualization. This matrix functioned as an advanced analytical resource, revealing detailed interactions between different energy contributors within the AMP‐resin complexes. Every cell in the correlation matrix displays a correlation coefficient value. The numerical value measured how strongly and in which direction the two variables were related to each other in the binding energy and each energy component. The range of correlation coefficients extended from −1 to 1, where positive figures revealed a positive correlation between variables, negative figures revealed a negative correlation, and values close to zero demonstrated minimal correlation between variables. A correlation coefficient of 1 indicated a perfect positive correlation, showing exact parallel increases in both analyzed variables, and a correlation coefficient of −1 demonstrated a perfect negative correlation, which meant that decreases in one variable exactly matched increases in another variable. A correlation coefficient of 0 demonstrated that there was no linear relationship between the analyzed variables. Analysis of correlation coefficients provided a valuable understanding of how AMPs interact with resins. The substantial positive correlation coefficient for van der Waals energy at 0.79 indicated that higher van der Waals energy values led to increased binding energy. The analysis showed that van der Waals interactions played a crucial role in stabilizing the complexes by contributing to their stability and affinity. Noncovalent van der Waals forces emerge between atoms and molecules because of changes in electron distribution within the particles. The significance of these interactions increases when molecules come into proximity, yet cannot form chemical bonds (Hermann et al. 2017; Al‐Hamdani and Tkatchenko 2019). Van der Waals interactions provide the necessary support for AMP‐resin complexes to retain their structural stability and functional performance. AMP molecules must align optimally and orient correctly on resin surfaces for specific interactions to optimize their antibacterial effectiveness within dental products. Dental resin composites benefit from improved stability through van der Waals forces, which extend their lifespan and toughness, and result in superior clinical performance.

The analysis revealed that electrostatic energy had a positive correlation coefficient of 0.28, indicating its beneficial role in complex stability; however, its influence remained weaker than that of van der Waals interactions. Research has indicated that electrostatic forces affect binding affinity, yet their strength is lower than that of van der Waals interactions. The attraction or repulsion between charged particles, including ions and polar molecules, produces electrostatic interactions, which are determined by the spatial distribution of charges in the molecules (Hattori et al. 2013; Hurd 1999). AMP‐resin complexes develop electrostatic forces through the interaction of charged or polar functional groups between the AMP molecules and resin surfaces. The moderately positive correlation coefficient (0.39 for desolvation energy underlined the importance of desolvation in enhancing AMP‐resin complex stability. The process of desolvation involves removing solvent molecules at the interface between AMP and resin, and this process demands energy (Cui et al. 2021). The energy used during desolvation plays a significant role in determining the total complex affinity, according to the observed correlation. The removal of solvent molecules at the interface during desolvation assists in creating strong intermolecular connections between AMP and resin, which in turn strengthens both stability and affinity characteristics of the complexes (Hussain and Maktedar 2023; Yuan 2023).

### 3.2 Molecular Docking Simulations of AMPs and Heme‐Binding Protein

**Figure 3:**
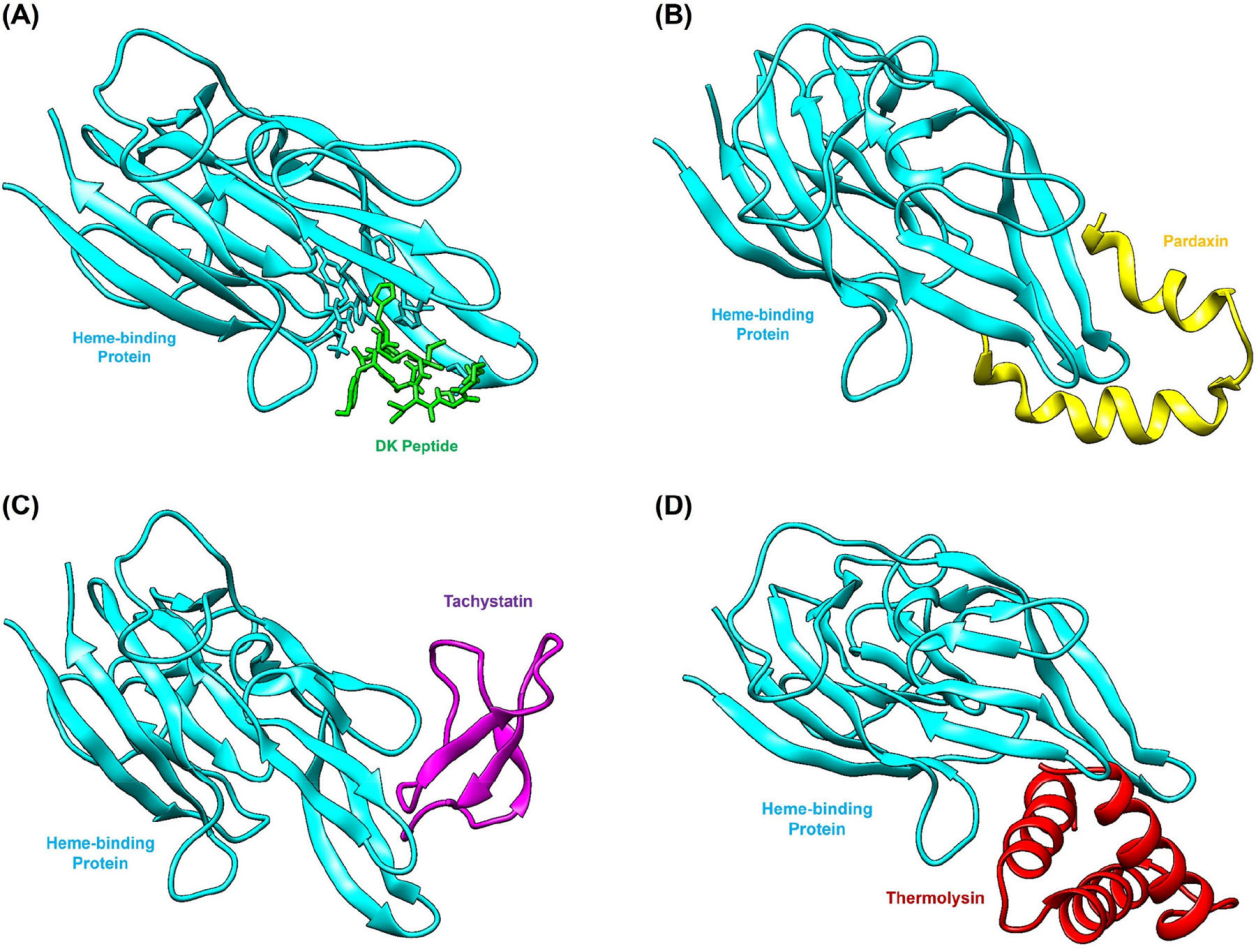
Molecular docking simulations revealed the optimal binding orientations for interactions between the heme‐binding protein and AMP. (A) Heme‐binding Protein:DK peptide (standard inhibitor) (B) Heme‐binding Protein:Pardaxin complex. (C) Heme‐binding Protein:Tachystatin. (D) Heme‐binding Protein:Thermolysin complex.

The three leading AMPs identified as Pardaxin, Tachystatin, and Thermolysin were further investigated to assess their binding interactions with a heme‐binding protein after initial dental resin composite results showed promise. This evaluation examined how these AMPs interact with important biological proteins to determine their potential binding abilities and interaction patterns, to assess their wider applications and functionality (Figure 3). Molecular docking simulations of the AMP‐heme‐binding protein complex revealed crucial details about their binding affinities and interaction dynamics. Table 4 details the comprehensive simulation results that display important parameters, including the HADDOCK score and various energy metrics such as binding energy, van der Waals energy, electrostatic energy, and desolvation energy, together with RMSD. Among all tested AMPs, the Pardaxin‐heme‐binding protein complex showed superior binding interactions with a HADDOCK score of −120.3 a.u. and a binding energy of −10.9 kcal/mol. The complex demonstrated strong stability through significantly favorable van der Waals and electrostatic energies, indicating robust noncovalent interactions. The docking results demonstrated high reliability, as the RMSD value of 1.0 Å showed significant structural similarity between the predicted and optimal conformations.

**Table 4:** Molecular docking results of the top five dental resin composites:antimicrobial peptide complexes.

Heme‐binding Protein:AMP Complex	HADDOCK score (a.u.)	Binding energy (kcal/mol)	Van der Waals energy	Electrostatic energy	Desolvation energy	RMSD
**Standard inhibitor**						
Heme‐binding Protein:DK peptide	−71.8 + /− 2.8	−7.5	−36.5 + /− 2.1	−77.1 + /− 9.2	−22.1 + /− 1.2	1.9 + /− 0.2
**Antimicrobial peptides (AMPs) complexes**						
Heme‐binding Protein:Pardaxin	−120.3 + /− 0.9	−10.9	−63.7 + /− 1.2	−132.3 + /− 19.9	−34.7 + /− 3.1	1.0 + /− 0.2
Heme‐binding Protein:Tachystatin	−100.1 + /− 1.7	−11.9	−55.6 + /− 2.6	−170.7 + /− 9.2	−12.0 + /− 1.9	1.3 + /− 0.1
Heme‐binding Protein:Thermolysin	−99.1 + /− 3.1	−10.2	−53.2 + /− 2.8	−189.4 + /− 11.5	−9.1 + /− 0.9	1.2 + /− 0.7

The Tachystatin‐heme‐binding protein complex formed strong binding interactions, as evidenced by its HADDOCK score of −100.1 a.u. and a binding energy of −11.9 kcal/mol. Substantial electrostatic interactions characterized the complex, as demonstrated by the high electrostatic energy of −170.7 kcal/mol. The docking prediction accuracy was confirmed by the RMSD value of 1.3 Å which demonstrated strong alignment between the predicted and optimal conformations. The Thermolysin‐heme‐binding protein complex demonstrated positive binding interactions, as evidenced by its HADDOCK score of −99.1 a.u. and a binding energy of −10.2 kcal/mol. The stability of this complex is enhanced by significant van der Waals and electrostatic interactions. The predicted and optimal conformations demonstrated a tight fit, as shown by an RMSD value of 1.2 Å, thus validating the docking simulation precision.

Table 5 presents comprehensive details about the intermolecular contacts (ICs) and noninteracting surface (NIS) areas from the heme‐binding protein complexes with both the standard antagonist (DK peptide) and AMPs. The research data indicated the precise types and quantities of intermolecular contacts that involved charged‐charged, charged‐polar, charged‐apolar, polar‐polar, polar‐apolar, and apolar‐apolar interactions. The Pardaxin‐heme‐binding protein complex was composed of three charged‐charged, four charged‐polar, 17 charged‐apolar, 19 polar‐apolar, and 20 apolar‐apolar interactions. The combination of multiple interaction types and the surface areas without interactions measuring 26.16 Å² for charged surfaces and 41.28 Å² for apolar surfaces demonstrated how the binding interactions are both extensive and complex. The Tachystatin‐heme‐binding protein complex formed six charged‐charged interactions, whereas six charged‐polar interactions occurred along with 25 charged‐apolar interactions, 3 polar‐polar interactions, 17 polar‐apolar interactions, and 18 apolar‐apolar interactions. The charged regions showed noninteracting surface areas of 27.84 Å², while the apolar regions had 35.23 Å², which demonstrates how various intermolecular contacts contribute to the stability of the complex. The Thermolysin‐heme‐binding protein complex formed four charged‐charged, nine charged‐polar, 25 charged‐apolar, two polar‐polar, 11 polar‐apolar, and 28 apolar‐apolar interactions. The Noninteracting surface areas measured 27.32 Å² for charged regions and 37.16 Å² for apolar regions, demonstrating the significant role of noncovalent interactions in complex stabilization.

**Table 5:** Intermolecular contacts and noninteracting surface areas of heme‐binding protein complexes with standard inhibitors and antimicrobial peptides.

Heme‐binding Protein:AMP Complex	ICs charged‐charged	ICs charged‐polar	ICs charged‐apolar	ICs polar‐polar	ICs polar‐apolar	ICs apolar‐apolar	NIS charged	NIS apolar
**Standard inhibitor**								
Heme‐binding Protein:DK peptide	5	1	17	0	1	8	29.93	36.05
**Antimicrobial ppeptides (AMPs) complexes**								
Heme‐binding Protein:Pardaxin	3	4	17	0	19	20	26.16	41.28
Heme‐binding Protein:Tachystatin	6	6	25	3	17	18	27.84	35.23
Heme‐binding Protein:Thermolysin	4	9	25	2	11	28	27.32	37.16

Abbreviations: ICs, Number of intermolecular contacts; NIS, Noninteracting surface.

### 3.3 Molecular Dynamics (MD) Simulation

**Figure 4:**
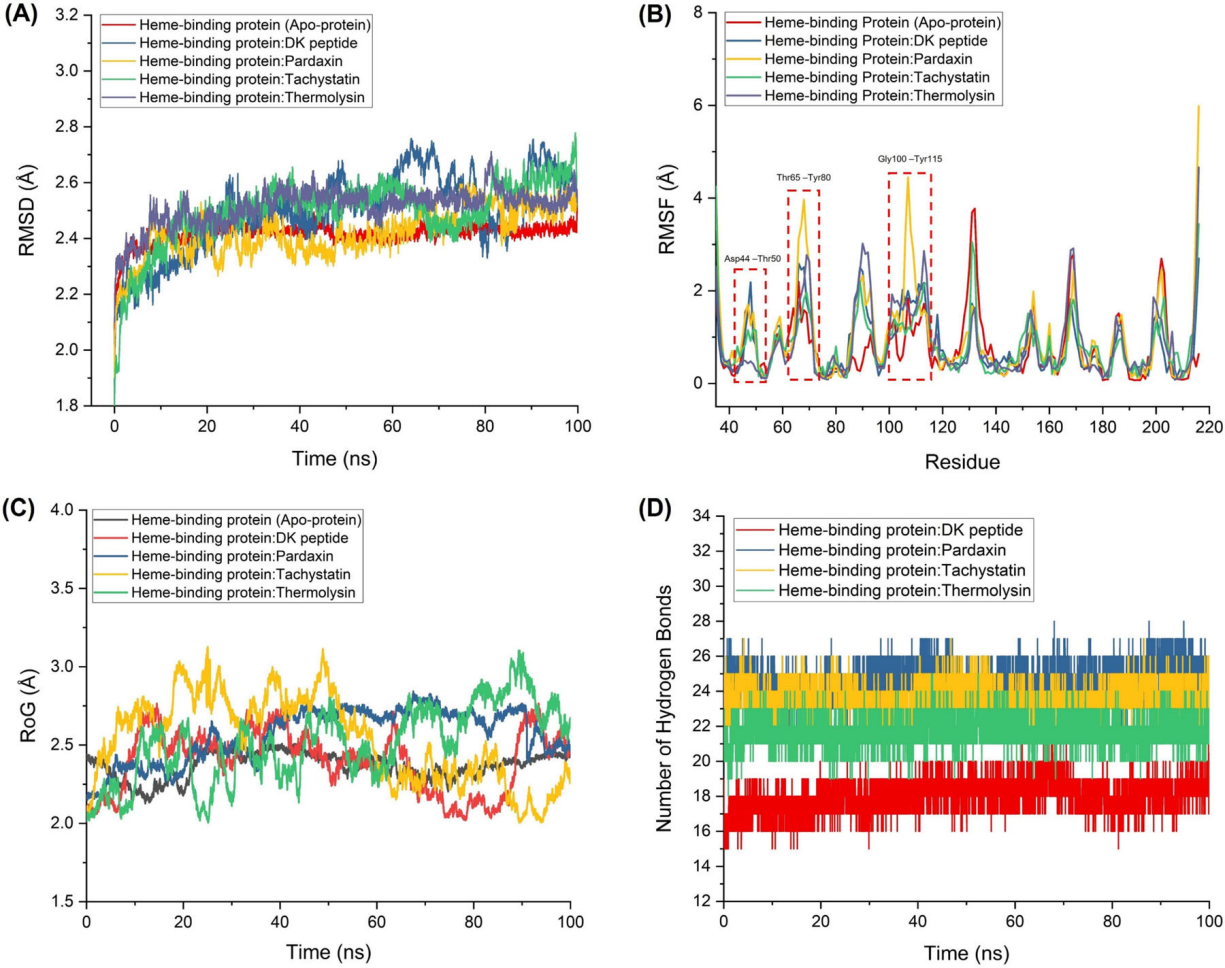
Molecular dynamics (MD) simulations of complexes formed between heme‐binding proteins and AMPs included several key parameters: (A) root mean square deviation (RMSD) assessed structural stability, (B) root mean square fluctuation (RMSF) depicted residue flexibility, (C) radius of gyration (RoG) illustrated structural compactness, and (D) number of hydrogen bonds highlighted intermolecular interactions.

MD simulation findings provide a complete understanding of how protein‐protein complexes between heme‐binding proteins and AMPs behave. During the 100 ns simulation, the heme‐binding protein showed consistent structural stability, as demonstrated by RMSD values between 2.417 and 2.513 Å, with no significant fluctuations (Figure 4A). Heme‐binding protein interactions between the standard inhibitor and AMPs remained consistent throughout the simulation period. RMSD measurements, which demonstrate how protein structures deviate from their original conformations, show distinct differences between the various complexes. The complex of the heme‐binding protein‐DK peptide standard inhibitor showed a greater average RMSD of 2.495 Å versus 2.417 Å for the apo‐protein alone. The binding of the standard inhibitor leads to a moderate increase in structural flexibility, which indicates that conformational changes are needed for effective binding. The AMP complexes formed with Pardaxin, Tachystatin, and Thermolysin demonstrated elevated average RMSD values between 2.423 and 2.512 Å when compared with the apo‐protein structure. These variations indicate multiple potential shifts in protein dynamics and structural changes triggered by AMP binding.

RMSF analysis in MD simulations helped to measure the flexibility of individual amino acid residues in the heme‐binding protein. The RMSF values computed for the entire protein spanned from 0.759 to 0.999 Å, showing that the protein exhibits moderate flexibility across its structure. Through this analysis, researchers gained important knowledge about residue mobility and flexibility, which led to an improved understanding of their functional roles in protein structure (Craveur et al. 2015). Analysis of the top‐performing AMP complex interaction with the heme‐binding protein showed significant hydrogen bond disruptions with specific residues, especially in regions containing the amino acid residues Asp44 Thr50, Thr65 Tyr80, and Gly100 Tyr115 (Figure 4B). These regions serve as active binding sites of the heme‐binding protein, which functions as the target receptor. Breaking hydrogen bonds in these essential sites causes residue motions to rise beyond the levels seen in the unbound apoprotein complex. The binding of the top‐performing AMPs to the heme‐binding protein promoted enhanced movement and flexibility of these residues. The conformational landscape of a protein changes dynamically because new interactions form while existing ones break, leading to increased flexibility. The top‐performing AMPs generate increased flexibility patterns that match those of the DK peptide, which serves as a known standard inhibitor. AMPs operate in a manner like that of the DK peptide, suggesting their potential effectiveness as inhibitors. RMSF data demonstrated that AMP binding to the heme‐binding protein triggers increased flexibility in essential areas, which could explain its inhibitory activity mechanism. Research into these dynamics is critical for the development of AMPs with enhanced binding affinity and specificity through rational design. These peptides can regulate protein functionality by targeting regions that enhance flexibility while breaking vital interactions, thereby providing new opportunities for therapeutic use.

MD simulation results showed that the radius of gyration (RoG) reflected protein structural compactness and expansion levels (Sanusi and Lobb 2022). This study examined RoG values for heme‐binding protein complexes to reveal the impact of various AMPs on protein structural integrity and compactness. The mean RoG values for heme‐binding protein complexes ranged from 2.371 to 2.564 Å, as shown in Figure 4C. The observed range reflects how protein compactness changes depending on the peptide binding. The standard antagonist complex containing the DK peptide had a RoG value of 2.410 Å. Researchers used this recorded value to gauge the compactness of the protein when it binds to the DK peptide. The AMP complexes displayed RoG values that were moderately elevated, between 2.510 and 2.564 Å. AMP complexes exhibit higher RoG values, indicating that they form slightly expanded or less compact structures than standard antagonists. AMPs exhibit distinct binding modes and interaction patterns, which lead to different levels of compactness in their heme‐binding proteins. The strength and specificity of the interaction between the heme‐binding protein and interacting peptides are indicated by the number of hydrogen bonds formed between them. The stability of the protein‐peptide complex depends on hydrogen bonding, which also plays an essential role in enhancing the binding affinity. Heme‐binding protein: The DK peptide complex established 18 hydrogen bonds, which provided the standard for comparison. The AMP complexes formed between 21 and 25 hydrogen bonds, which exceeded the number found in the other complexes. Notably, the heme‐binding protein pardaxin complex established the maximum number of hydrogen bonds, totaling 25. This was followed closely by the heme‐binding protein:Tachystatin complex, which exhibited 23 hydrogen bonds, while the heme‐binding protein Thermolysin complex exhibited 21 hydrogen bonds, as shown in Figure 4D. AMP complexes showed more hydrogen bonds, indicating stronger and more precise interactions with the heme‐binding protein.

### 3.4 Molecular Mechanics/Poisson–Boltzmann Surface Area (MM/PBSA) Calculations

Using MM/PBSA methods, researchers have determined the binding free energy of heme‐binding protein complexes with high‐performing AMPs and DK peptides. Binding free energy is a crucial indicator for evaluating protein complex stability and interaction strength based on thermodynamic favorability. DK peptide served as the standard inhibitor for the heme‐binding protein complex and yielded an average Δ*G*_binding of −32.52 kcal/mol over three computational runs. The binding free energy values measured for the complex were −32.47, −32.74, and −32.34 kcal/mol. The average free energy change value reflects the stability and intensity of the interaction between the heme‐binding protein and DK peptide. The binding strength and stability increased when Δ*G*_binding became more negative (Table 6).

**Table 6:** The average binding free energy (Δ*G*_binding_) for the heme‐binding protein complexes was reported as the standard deviation in kcal/mol units, as determined by MM/PBSA calculations.

Complex	MM/PBSA calculation results Δ*G*_binding_ (kcal/mol)			Average (kcal/mol)
	I	II	III	
**Standard inhibitor**				
Heme‐binding protein:DK peptide	−32.47	−32.74	−32.34	−32.52
**Antimicrobial peptides (AMPs) complexes**				
Heme‐binding protein:Pardaxin	−66.31	−67.15	−63.30	−65.58
Heme‐binding protein:Tachystatin	−48.86	−48.39	−48.87	−48.71
Heme‐binding protein:Thermolysin	−39.14	−40.06	−40.55	−39.92

The binding free energies of the AMP complexes varied, demonstrating their diverse interaction strengths with the heme‐binding protein. Heme‐binding protein: The Pardaxin complex demonstrated an average Δ*G*_binding value of −65.58 kcal/mol. The observed Δ*G*_binding values stood at −66.31, −67.15, and −63.30 kcal/mol individually. The substantial negativity of average Δ*G*_binding reflects a strong and stable binding interaction, demonstrating the effectiveness of pardaxin in forming a stable complex with the heme‐binding protein. The heme‐binding protein: The Tachystatin complex displayed an average Δ*G*_binding of −48.71 kcal/mol, which consisted of individual values −48.86, −48.39, and −48.87 kcal/mol. The average Δ*G*_binding value proved to be less negative when compared to pardaxin, yet it demonstrates a strong interaction with the heme‐binding protein. The heme‐binding protein: The Thermolysin complex exhibited an average Δ*G*_binding of −39.92 kcal/mol, which resulted from individual measurements of −39.14, −40.06, and −40.55 kcal/mol. This average Δ*G*_binding value is lower than that of the DK peptide, but higher than that of both Pardaxin and Tachystatin, indicating moderate binding strength. Analysis through MM/PBSA revealed differentiation in binding strengths between AMPs and the heme‐binding protein while marking pardaxin as the strongest binder. AMP complexes with lower Δ*G*_binding values especially in Pardaxin demonstrated enhanced binding affinities and exhibited increased stability compared to the standard inhibitor. Research data clearly show that AMPs function as effective therapeutic agents against heme‐binding proteins and exceed traditional inhibitors in performance.

## 4 Discussion

Through molecular simulations, we investigated how AMPs interact with dental resin composites to determine methods for incorporating AMPs into dental materials. Our simulations demonstrated that various AMPs bind with dental resin composites through several mechanisms, including hydrophobic forces, hydrogen bonds, and salt bridges. AMP‐resin composite performance relies on AMP‐resin composite interactions, while many AMP‐resin combinations show exceptional stability with strong binding affinity. Bis‐GMA formed strong binding interfaces with Thermolysin through multiple hydrophobic interactions and salt bridge formation. The strong and precise binding of Pardaxin to EBPADMA through hydrophobic interactions and hydrogen bonds shows its potential effectiveness in dental resin composites. Past research findings show that peptide‐resin composite stability relies predominantly on electrostatic interactions and hydrophobic forces. Studies have shown that hydrophobic interactions significantly contribute to the stability of peptide‐resin composites, resulting in better dental application performance (Moussa et al. 2019; Delaviz et al. 2014). Research has shown that AMPs interact differently with resin composites, with some interactions resulting in stronger and more extensive bonds. Multiple hydrogen bonds and hydrophobic interactions between Tachystatin and HEMA indicated a stable and well‐defined binding interface. Other studies support this finding by showing that peptides with numerous hydrogen bonds demonstrate increased binding strength and stability with biomaterials (Zhou et al. 2024; Gan et al. 2021).

From an applicative perspective, the present results suggest that Pardaxin and Tachystatin could serve as strong candidates for direct incorporation into dental composites due to their high binding affinity and molecular stability. Thermolysin, although showing the lowest binding energy (Δ*G* = −39.92 kcal/mol), remains relevant for further investigation because of its distinctive binding configuration and the presence of stabilizing hydrophobic and electrostatic contacts within the resin matrix. These characteristics indicate that Thermolysin may contribute synergistically to antimicrobial activity, particularly under different environmental or compositional conditions, such as variations in resin formulation or oral pH. Therefore, retaining Thermolysin in the analysis broadens the understanding of diverse peptide‐material interaction behaviors, which is valuable for the rational design of multifunctional dental composites.

This study examined how the best‐performing AMPs, including Pardaxin, Tachystatin, and Thermolysin, interact with a heme‐binding protein after their AMP‐resin interaction evaluation. This study evaluated how these AMPs can be used in different biological settings by studying their interactions with important biological proteins. Docking simulation results demonstrated that each of the three AMPs established strong binding interactions with the heme‐binding protein. Pardaxin established its strongest binding interactions through substantial van der Waals forces and electrostatic interactions, which enhanced its stability. Tachystatin maintains a strong binding through electrostatic interactions. Thermolysin displayed a strong binding potential, even though it fell short of being as favorable as other proteins. Nonetheless, its balanced interaction profile suggests that it may provide a moderate yet steady antimicrobial contribution when integrated within composite systems, potentially reducing bacterial resistance risks through multi‐mechanistic action.

Our results on peptide‐protein interactions confirm previous studies that identified van der Waals and electrostatic forces as key factors in determining binding affinity and stability. Studies have revealed that peptide‐protein complexes achieve enhanced stability and effectiveness through the combination of van der Waals and electrostatic interactions, according to our AMP‐heme‐binding protein interaction data (Zhu et al. 2021; Hosseinpour et al. 2020). The study of intermolecular contacts provided clear insights into the interaction mechanisms between AMPs and their respective heme‐binding proteins. The binding affinity of pardaxin was heightened because of its multiple interaction types, including charged‐charged, charged‐polar, and apolar‐apolar interactions. Scientific studies demonstrated that different noncovalent interactions significantly contribute to peptide‐protein complex stabilization while showing that binding stability and efficacy are enhanced through diverse interaction mechanisms (Adhav and Saikrishnan 2023). MD simulations demonstrated dynamic behavior in AMP‐heme‐binding protein complexes. AMP binding to heme‐binding proteins triggers essential conformational changes through alterations in both protein stability and regional flexibility. Multiple research studies have confirmed that peptide interactions cause target proteins to undergo structural changes that enhance flexibility and alter functional states. The research illustrates how peptide binding requires conformational adjustments to reveal its functional impacts (Gupta et al. 2022; Al Qaraghuli et al. 2020). These findings demonstrate that AMPs can enhance the antibacterial properties of dental resin composites by forming stable interfaces with both resin matrices and bacterial protein targets. This dual‐action mechanism (material integration and biofilm inhibition) supports the translational relevance of in silico modeling as a preliminary step for developing next‐generation bioactive dental materials.

## 5 Limitations and Clinical Considerations

Through molecular docking simulations, we gained valuable insights that must be considered along with numerous limitations. High‐quality input data and precise force field accuracy are essential components for accurate molecular docking predictions. The static nature of models for AMPs and dental resin composites limits our docking predictions by failing to capture the dynamic elements of biological interactions. The restriction shows its importance in complex systems because binding affinity and stability require conformational flexibility. Research has shown that docking simulations that apply the rigid body approach may simplify interactions too much, resulting in less accurate predictions for binding affinities and mechanism details (Pantsar and Poso 2018; Meng et al. 2011). In this study, we utilized the HADDOCK docking tools to explore AMP–resin monomer interactions; however, it is important to note that HADDOCK primarily relies on rigid‐body docking, which may not fully account for conformational flexibility and solvent effects. This limitation can lead to potential inaccuracies in estimating true binding energies and interaction orientations, particularly for systems involving flexible peptides like AMPs. Therefore, while HADDOCK provided valuable preliminary insights into the binding patterns of AMPs with resin monomers, the results should be interpreted cautiously and complemented with more advanced flexible docking or dynamic simulations in future research.

Our simulations reveal details about peptide‐resin interactions and AMP binding to heme‐binding proteins, but fail to consider every biological element that affects the performance and safety of these materials in clinical applications. The prediction of interactions depends solely on a single docking method, which restricts the scope of our study. The results of the docking algorithms differed according to their scoring functions and sampling methods. Our findings will become more reliable if verified using various docking methods and experimental techniques.

Dental resin composites with added AMPs demonstrate enhanced effectiveness in controlling microbial growth within dental applications. Clinical application of these findings needs careful analysis of multiple important aspects. Before clinical application can proceed, researchers need to complete extensive safety and biocompatibility evaluations of dental material‐integrated AMPs. The antimicrobial properties of AMPs necessitate complete in vitro and in vivo testing to determine their cytotoxic potential and harmful impacts on oral tissues. Research has shown that AMPs at high doses exhibit cytotoxic properties, thereby emphasizing the importance of precise dosage optimization and comprehensive safety testing (Ki et al. 2023; Ma et al. 2024). Therefore, while the computational results presented here are promising, they should not be interpreted as definitive clinical solutions. Extensive in vitro and in vivo investigations remain essential to validate AMP efficacy, biocompatibility, and long‐term behavior within dental resin matrices.

The effectiveness of AMPs in clinical applications depends on their long‐term stability and release kinetics when incorporated into the resin composites. Research on the performance of AMP‐resin composites across different oral hygiene and functional conditions is necessary to confirm their consistent antimicrobial effectiveness. Development of resistance to AMPs is an important clinical consideration. There is a risk that bacteria could develop resistance to AMPs over time, like traditional antibiotics, which could reduce their effectiveness. The long‐term effectiveness of AMP‐based dental materials depends on proper AMP treatment and research on resistance patterns. Therefore, researchers need to assess the economic benefits of adding AMPs to dental resin composites. The production and integration of AMPs into dental materials incurs additional costs despite their powerful antimicrobial capabilities. To assess the practicality of adopting AMP‐enhanced composites in clinical settings, researchers must conduct a cost–benefit analysis comparing these materials with traditional dental materials.

## 6 Conclusion and Future Works

Our study shows that dental resin composites gain improved antimicrobial properties when they contain AMPs like Pardaxin, Tachystatin, and Thermolysin. Pardaxin displays broad antibacterial activity against multiple pathogens, while Tachystatin and Thermolysin use targeted mechanisms for effective biofilm management and resistance reduction. These findings highlight the strong potential of AMPs as promising candidates for enhancing the antimicrobial performance of dental composites. However, further experimental validation is essential to translate these computational insights into clinical applications. In particular, extensive in vitro and in vivo investigations are needed to confirm the biological safety, efficacy, and long‐term stability of AMP‐functionalized composites under physiological conditions. Research indicates that dental materials combined with AMPs require complete validation through laboratory and live organism studies to establish their safety and durability. Future studies should also focus on understanding how saliva components, pH fluctuations, and enzymatic degradation influence AMP activity within the composite matrix. Subsequent research should examine how biological interaction dynamics and biological fluids impact AMP activity to understand study limitations. Clinical research must examine how different AMP concentrations interact with release kinetics and resistance development. The combination of several docking methods with experimental approaches will improve prediction validation and enhance our knowledge of AMP‐resin interactions. Subsequent research should evaluate the cost‐effectiveness of AMP incorporation in dental composites while exploring their broader applications in biomedical areas that require antimicrobial capabilities.

## Author Contributions

**Ravinder S. Saini:** conceptualization, methodology, original draft preparation, supervision and project administration. **Doni Dermawan:** conceptualization, methodology, data curation, formal analysis, investigation, resources, writing, reviewing, and editing. **Abdulkhaliq Ali F. Alshadidi:** writing, reviewing, and editing. **Rayan Ibrahim H. Binduhayyim:** investigation, resources. **Rajesh Vyas:** data curation, formal analysis. **Fahad Hussain Alhamoudi:** original draft preparation. **Sunil Kumar Vaddamanu:** investigation, resources. **Mohammed Saheer Kuruniyan:** original draft preparation. **Lujain Ibrahim N. Aldosari:** data curation, formal analysis, writing, reviewing, and editing. **Artak Heboyan:** validation, formal analysis. writing, reviewing, and editing, supervision, project administration.

## Ethics Statement

The authors have nothing to report.

## Conflicts of Interest

None declared.

## Supporting information

Supplemental material revised.

Supplemental material revised.

## Data Availability

The data is available to the corresponding author upon request.
